# Demographics of Scorpion Sting in Iran; a Cross Sectional Study

**DOI:** 10.22037/emergency.v5i1.18276

**Published:** 2017-08-21

**Authors:** Babak Mahshidfar, Hamed Basir Ghafouri, Mohammad Reza Yasinzadeh, Mani Mofidi, Mahdi Rezai, Davood Farsi, Saeed Abbasi, Peyman Hafezimoghadam

**Affiliations:** Emergency Medicine Management Research Center, Rasoul-e-Akram Hospital, Iran University of Medical Sciences, Tehran, Iran.

**Keywords:** Iran, scorpion stings, epidemiologic studies, emergency service, hospital

## Abstract

**Introduction:**

Scorpion sting is an important public health problem in some countries, including Iran. This study aimed to describe the demographics of a large number of these victims in some endemic areas of Iran.

**Methods:**

This cross-sectional study evaluated baseline characteristics, clinical findings, management, and disposition of scorpion stung cases in 26 cities of 4 provinces in the southwest quarter of Iran, during one year.

**Results:**

3008 cases of scorpion sting with mean age of 27.07 ± 16.58 years were studied (51.3% female). The mean time from sting to hospital was 1.89 ± 1.04 hours. No first aid measures had been taken in 96.6% of cases. Lower (39.5%) and upper (35.7%) extremities were stung most frequently. Midnight to 6:00 am was the period of time most of stings occurred (34.2%). Local pain (77.2%) and erythema (63.5) were among the most common signs and symptoms. 2026 (67.3%) victims had been discharged; 326 (10.8%) were admitted or referred to other hospitals and 5 (0.2%) cases died.

**Conclusion:**

It seems that demographic characteristics of scorpion sting in Iran are not so different from those reported from other sites of the world, as signs and symptoms of local and systemic envenomations. Victims, companions, and healthcare providers perform many futile and maybe harmful measures and there is a need to educate all about all of these details.

## Introduction

Scorpion sting is an important public health problem in some countries, including Iran. The scorpion envenomation mortality rate is estimated to be about 0.27% and some of the species could kill their victims in about 7 hours (1). At least 1 million scorpion stings are estimated to occur annually around the world, leading to more than 3250 deaths (2, 3). In Iran, scorpions have been classified in 2-3 families, 8-10 genera, 18-32 species, and 7-17 subspecies; and they have threatened a relatively large population in significant regions (4-7). Approximately 40000-50000 cases of scorpion sting have been recorded, annually in Iran, with about 19 deaths each year (8, 9). In Iran, there were 42,850 scorpion sting events reported with 14 deaths in 2005 compared to 45950 cases with 18 deaths in 2006. Khuzestan and Hormozgan are two Iranian provinces having lots of scorpion sting cases reported, annually (4, 10). The true incidence of scorpion sting events is unclear, because some cases do not request medical attention. Epidemiologic studies may improve the determinants of scorpion sting in order to plan and implement effective public health interventions. Recognition of clinical presentations and complications of scorpion sting is invaluable as it could light the way to therapeutic strategies. The other important aspect of encountering scorpions is that we should train skilled physicians who can accurately determine the type of scorpions and know the characteristics of scorpions endemic to an area. This strategy can lead to a rapid and correct selection of therapeutic modalities (11, 12). In this study we tried to describe demographics of a large population of these victims presenting to emergency departments (ED) of endemic areas in Iran.

## Method

***Study design and setting***

In this cross-sectional study, we evaluated the baseline characteristics, clinical findings, management, and disposition of scorpion stung cases in 26 cities of 4 provinces in the southwest quarter of Iran (Khuzestan, Kohkiluye Boyerahmad, Hormozgan, and Kerman), during April to December 2009. Our study protocol was approved by the ethics committee of Iran University of Medical Sciences (IUMS). All authors adhered to world medical association declaration of Helsinki, ethical principles for medical research involving human subjects.

***Participants***

All scorpion stung patients referring to the governmental clinics (both outpatients and those admitted) in the defined cities with available data on Hospital Information System (HIS), were included. There was not any sex or age limitation. Patients with missing data were excluded.

***Data collecting***

A comprehensive database including demographic information (sex, age, etc.), scorpion characteristics, time of sting, history of previous sting, site of body stung, clinical signs and symptoms, as well as prehospital and ED management (anti-venom, antibiotic, corticosteroid, etc.) and disposition of patients were filled by the physician in charge. 

***Statistical analysis***

Statistical analyses were performed with SPSS_ 21 and descriptive findings were presented as frequency and percentage or mean ± standard deviation.

## Results

***Baseline characteristics***

Data of 3008 cases of scorpion stings with mean age of 27.07 ± 16.58 (5 month- 99) years were collected (51.3% female). 1315 (45%) cases were in the age range of 20 -39 years. Tables 1 and 2 show the baseline characteristics of the studied patients. Most cases (71.6%) were recorded in Khuzestan province. The mean time from sting to hospital referral was 1.89 ± 1.04 hours and most victims had been referred to medical facilities within 1.5 hours following stings (44.1%). Only 7.7% of patients were visited after 6 hours following stings. No first aid measure had been taken in 96.6% of cases; among first aid measures taken, tight bandage of stung site had most frequently been done, followed by incision and suction of the site and analgesic use. Lower extremities were stung most frequently (39.5%), followed by upper extremities (35.7%). Midnight to 6:00 am was the period of time most of stings occurred (34.2%). In 51% of cases, the scorpion had been described as yellow and in 20.9% as black; in the rest of forms, color of scorpion had not been defined. 

***Clinical findings***

Clinical findings of patients are summarized in table 3. Pain (77.2%) and erythema (63.5%), tachycardia (3.6%), agitation (1.0%), mouth xerosis (5.6%), hematuria (1.6%) and rash (2.4%) were among the most common local, cardiopulmonary, neurologic, gastrointestinal, urologic, and skin problems, respectively. Urinalysis (U/A) was performed for 1688 (56.1%) cases; 21% of which had hematuria. 

***Management***

Polyvalent scorpion anti-venom had been prescribed for 2489 (82.7%) victims (87.3% intramuscular and 12.7% intravenous). 132 (5.3%) victims were given more than one vial of anti-venom, 35 (1.4%) of which experienced an acute hypersensitivity reaction; 17 cases were limited to skin. Skin test for anti-venom had been accomplished in 2383 (79.2%) of victims. 

The other medications prescribed included anti-histamines (46.9%), systemic corticosteroids (36.2%), analgesics (24.9%), anti-microbial (10.4%), atropine (0.4%), and anti-hypertensive (0.2%). 

***Disposition***

2026 (67.3%) victims had been recovered in EDs and were discharged; 326 (10.8%) had been admitted or referred to other hospitals. 5 (0.2%) cases died; all of them had been given anti-venom.

## Discussion

The majority of studies from Iran and other countries have reported that scorpion stings are more prevalent in summer, especially July (13-18). Our data showed that most of the stings recorded occurred during October, September, and November. 

**Table 1:** Baseline characteristics of studied patients

**Variables**	**Number (%)**
**Sex**	
Female	1523 (51.3)
Male	1443 (48.6)
**Age (year)**	
<20	994 (34.0)
20-39	1315 (45.0)
40-59	463 (15.8)
60-79	132 (4.5)
>80	19 (0.7)
**Pervious sting**	
Yes	1116 (49.7)
No	1129 (50.3)
**Time to hospital (hour)**	
<1.5	1328 (51.0)
1.5-3	457 (17.6)
3-6	584 (22.4)
>6	231 (8.9)
**Province**	
Khuzestan	1709 (71.6)
Kohkiluye Boyerahmad	488 (20.4)
Hormozgan	146 (6.1)
Kerman	45 (1.8)
**Season**	
Spring	29 (1.0)
Summer	1164 (40.6)
Autumn	1563 (54.5)
Winter	114 (4.0)
**Pre-hospital management**	
None	1339 (92.9)
Tourniquet	38 (2.6)
Analgesic	25 (1.7)
Wound sucking	24 (1.7)
Disinfection	9 (0.6)
Onion application	6 (0.4)

**Table 2:** Characteristics of stings

**Variables**	**Number (%)**
**Location**	
Upper extremities	1073 (38.4)
Lower extremities	1187 (42.5)
Head and neck	124 (4.4)
Trunk	411 (14.7)
**Time**	
0-6	1030 (44.9)
6-12	450 (19.6)
12-18	302 (13.2)
18-24	506 (22.1)
**Scorpion color**	
Yellow	1534 (59.9)
Black	629 (24.5)
Unknown	399 (15.6)

**Table 3:** Clinical findings of studied patients

**Variables**	**Number (%)**
**Local**	
Pain	2157 (77.2)
Erythema	1774 (63.5)
Muscular pain	165 (5.9)
Numbness	160 (5.7)
**Cardiopulmonary**	
Tachycardia	101 (3.6)
Bradycardia	23 (0.8)
Hypertension	29 (1.0)
Hypotension	10 (0.4)
Dysrhythmia	7 (0.3)
Respiratory distress	17 (0.6)
Wheezing	3 (0.1)
Pulmonary edema	1 (0.03)
**Neurologic**	
Agitation	28 (1.0)
Irritability	25 (0.9)
Paresthesia	24 (0.9)
Confusion	5 (0.2)
Seizure	5 (0.2)
Fasciculation	2 (0.1)
**Gastrointestinal**	
Mouth xerosis	156 (5.6)
Nausea and vomiting	119 (4.3)
Hyper salivation	34 (1.2)
Abdominal pain	19 (0.7)
Diarrhea	7 (0.3)
**Urologic**	
Hematuria	45 (1.6)
Urinary retention	9 (0.3)
Frequency	7 (0.3)
Priapism	2 (0.1)
**Skin**	
Rash	67 (2.4)
Anaphylaxis	6 (0.2)
Angioedema	4 (0.1)

The envenomation episodes had occurred almost equally in males and females confirming the results of most studies done before; although, certain studies showed a male preference and in one study from Brazil, victims were predominantly female (2, 6, 11, 13, 19-23). 

The 20-30 years age group was the most affected one, similar to the results reported by the other studies from Iran and other countries (2, 3, 6, 13, 23, 24). The time between sting and ED arrival was acceptable and similar to most previous studies (20, 23). 

In the current study, lower extremities followed by upper extremities were shown to be more commonly stung, compared with other parts of the body, in line with the results reported by Dehghani, Nejati, and Barros (6, 11, 23). It can be due to the fact that people of the studied regions wear unsecured shoes due to their low socio-economic status and children walk around with bare feet and play with stones.

Yellow scorpions were responsible for half of the stings; however, the color of the scorpions remained unknown in 20% of the cases. Other studies from Iran have reported similar result (11, 20). Two investigations in Turkey found that black scorpion sting events were more prevalent than others (25, 26).

On-scene managements were done for only 7% of the victims in our study. This finding was in contrast to a study that first aids were done in 95% of sting episodes (25). Ligation, immobilization, cutting, sucking, and cleaning of the site of sting were done mostly (13).

Studies have revealed that venom of scorpion stimulates the sympathetic system and increases blood pressure (hypertension) and heart rate (tachycardia) and induces cardiac dysrhythmias (18). It stimulates catecholamine release in body that leads to anxiety, agitation, tachycardia, hypertension, fever, and sweating. It also stimulates the parasympathetic system transiently causing urinary retention, sweating, and sialorrhea (27). 

In the current study, local signs and symptoms including pain and erythema were more common. Other frequent symptoms or signs were restlessness, tachycardia, dry mouth, hematuria, flank pain, and urticarial rash. These findings have been frequently mentioned in previous studies (13, 18). In 2002, a study reported 261 sting events in Birjand with clinical presentations including pain, paresthesia, erythema, and itching in 100% of cases. Agitation, sweating, and nausea were seen in 20% of them. No seizure, dysrhythmia, or hemodynamic instability were seen (18). In a study conducted in 2001-2011, 790 envenomation episodes were assessed in Qom province of Iran. The clinical presentations included pain at the site of envenomation (82%), local redness (9%), hypoesthesia and numbness (12.5%), and severe muscle pain (0.7%). In that study, 97% of victims did not show neurologic symptoms; positive neurologic symptoms had involved sympathetic (2%), parasympathetic (0.5%), and central nervous system (0.2%) (20). 

Barros studied 2283 scorpion sting victims in the years 2007-2012 in Brazil. The most frequent local manifestations were pain (96.14%), edema (30.35%), and paresthesia (19.92%). While generalized pains (1.66%), vagal manifestations (1.05%), and arterial hypotension (0.26%) were among the most frequent systemic clinical manifestations. Local pain was notified in almost 100% of the cases (23). In Saudi Arabia, 251 scorpion envenomed cases were reported in the years 1986-2000. 95% of victims had local pain and 78.3% had systemic presentations such as hypertension, hypersalivation, and sweating (14).

Management of scorpion sting includes fluid and electrolyte replacement, antibiotics in some cases, tetanus immunization, anti-venom, and analgesics (25). The use of anti-venom has been further challenged, because it could result in allergic complications. However, it is commonly believed that anti-venom therapy is the most effective treatment (28). In the current study, about 82% of patients received anti-venom. With regard to other studies done in Iran, anti-venom is used in the majority of scorpion sting episodes (2, 6, 20).

The mortality of scorpion sting was 0.2%, which is higher than those reported in previous studies. Studies from Turkey, Saudi Arabia, and Iran (Qom) reported no lethality (6, 13, 14, 20, 25). Dehghani et al. evaluated 418 scorpion sting cases in Khuzestan province in 2003 and the mortality rate was 0.05% (11). Bouaziz et al. found that the following factors correlated with a poor outcome in scorpion stings: age less than 5 years, fever more than 38.5°^C^, Glasgow coma score (GCS) equal to 8/15 or less, pulmonary edema, leukocytosis more than 25,000 cells/mm^3^, and blood urea level above 8 mmol/L (29). Others stated that factors such as victim’s age, weight, and health status, site of body stung, time of sting, and type of scorpion have important roles in severity of envenomation. Envenomation is more dangerous in both extremes of age and victims with lower weight (25, 30).

Scorpion envenomation is still a health problem in Iran and planning educational strategies for its prevention and management is necessary; we cannot overcome this hazard without optimal identification of the epidemiology of scorpions and the clinical manifestations of envenomation. Although the majority of sting events occurred in the south of the country, but this problem has extended to the capital, Tehran; 2.7% of mortalities in Loghman Hakim poison center is related to scorpion sting (31).

Obviously, the results can provide the health care providers with valuable information about the similarities and differences of such complications compared with other places in the world. It would guide us in designing protocols and guidelines to evaluate and manage the victims.

***Limitation***

Old data is an important limitation of the present study but considering the large sample size, the finding could be representative of the demographic features of scorpion sting during that time period.

## Conclusion

It seems that demographic characteristics of scorpion sting in Iran are not so different from those reported from other sites of the world, as signs and symptoms of local and systemic envenomations. Victims, companions, and healthcare providers perform many futile and maybe harmful measures and there is a need to educate all about all of these details.
